# IL-21 Stimulates the expression and activation of cell cycle regulators and promotes cell proliferation in EBV-positive diffuse large B cell lymphoma

**DOI:** 10.1038/s41598-020-69227-0

**Published:** 2020-07-23

**Authors:** Yuxuan Wang, Chengcheng Wang, Xiyunyi Cai, Chang Mou, Xueting Cui, Yingying Zhang, Feng Ge, Hao Dong, Yuanyuan Hao, Lei Cai, Shuting Wu, Chenjie Feng, Jiamin Chen, Jianyong Li, Wei Xu, Lei Fan, Weijia Xie, Yue Tong, Harvest Feng Gu, Liang Wu

**Affiliations:** 1 Jiangsu Key Laboratory of Drug Screening, China Pharmaceutical University, Nanjing, 210009 China; 2 Department of Hematology, The First Affiliated Hospital of Nanjing Medical University, Jiangsu Province Hospital, Collaborative Innovation Center for Cancer Personalized Medicine, Nanjing, 210029 China; 3 State Key Laboratory of Natural Medicines, China Pharmaceutical University, Nanjing, China; 4 Department of Medicinal Chemistry, China Pharmaceutical University, Nanjing, China; 5 Jiangsu Key Laboratory of Druggability of Biopharmaceuticals, China Pharmaceutical University, Nanjing, China; 6 School of Life Science and Technology, China Pharmaceutical University, Nanjing, China; 7 School of Basic Medicine and Clinical Pharmacy, China Pharmaceutical University, Nanjing, China

**Keywords:** Cellular signalling networks, Tumour immunology, Tumour virus infections, B-cell lymphoma, Cancer microenvironment

## Abstract

The clinical features of EBV-positive diffuse large B cell lymphoma (DLBCL) indicate a poorer prognosis than EBV-negative DLBCL. Currently, there is no efficacious drug for EBV-positive DLBCL. The cytokine interleukin-21 (IL-21) has been reported to be pro-apoptotic in DLBCL cell lines and is being explored as a new therapeutic strategy for this type of lymphomas. However, our previous studies showed that IL-21 stimulation of EBV-positive DLBCL cell lines leads to increased proliferation. Here, analysis of a rare clinical sample of EBV-positive DLBCL, in combination with a NOD/SCID mouse xenograft model, confirmed the effect of IL-21 on the proliferation of EBV-positive DLBCL cells. Using RNA-sequencing, we identified the pattern of differentially-expressed genes following IL-21 treatment and verified the expression of key genes at the protein level using western blotting. We found that IL-21 upregulates expression of the host *MYC* and AP-1 (composed of related Jun and Fos family proteins) and STAT3 phosphorylation, as well as expression of the viral LMP-1 protein. These proteins are known to promote the G1/S phase transition to accelerate cell cycle progression. Furthermore, in NOD/SCID mouse xenograft model experiments, we found that IL-21 treatment increases glucose uptake and angiogenesis in EBV-positive DLBCL tumours. Although more samples are needed to validate these observations, our study reconfirms the adverse effects of IL-21 on EBV-positive DLBCL, which has implications for the drug development of DLBCL.

## Introduction

Diffuse large B cell lymphoma (DLBCL) accounts for 30% of adult non-Hodgkin's lymphoma (NHL) and is the most common type of malignant lymphoma. Epstein-Barr virus (EBV)-positive DLBCL is a newly defined subgroup of DLBCL that refers to the proliferation of B cell clones carrying EBV^1^. The 2008 World Health Organization classification included “Elderly EBV-positive DLBCL” as a temporary entity. However, EBV-positive DLBCL is now increasingly recognised in younger patients, with a broader morphological profile and better survival outcome than originally thought, resulting in the replacement of the name "elderly EBV-positive DLBCL" with “EBV-positive DLBCL”^1^. Approximately 10% of all patients with DLBCL present with EBV positivity^2–4^. The clinical features of EBV-positive DLBCL and EBV-negative DLBCL are quite different, and the prognosis of EBV-positive DLBCL is poorer^3–8^. Currently, there is no efficacious drug for EBV-positive DLBCL.

EBV is associated with a variety of human tumours^9^. EBV infects adults in almost all parts of the world and is one of the most successful viruses in humans^10^. EBV uses different latency types to drive the transition of infected B cells from resting B cells to memory cells. Type III latency, which results from expression of the full set of viral latency proteins, can be detected in EBV-infected B cells in vitro (conditional lymphoblastoid cell lines, LCLs) and in patients with infectious mononucleosis or a subset of posttransplant lymphoproliferative disorders (PTLDs); whereas type II latency (expressing EBNA-1 together with LMPs and EBER) are detected in classical Hodgkin lymphoma and a subset of PTLD^9,11^. EBV-positive DLBCL shares a similar EBV latency patterns (type II or III) with PTLD^12^.

A variety of cytokines are known to modulate the expression of EBV latency genes^9,13,14^. IL-21 induces LMP-1 expression, as well as activation of the host STAT3, in a type I latency Burkitt lymphoma cell line and in a conditional LCL^9^. This common gamma chain cytokine is an effective B cell activator and plasma cell differentiation inducer produced by CD4 + T cells^15^. The effect that treatment with this cytokine has on EBV-positive lymphoma cell lines seems to depend on the type of EBV latency programme. Specifically, IL-21 treatment of type I latency Burkitt lymphoma cells inhibits cell proliferation, whereas in type III latency LCL and Burkitt lymphoma cell lines it induces plasma cell differentiation^9^. Similarly, we found that regulation of EBV latency gene expression by IL-21 in five DLBCL-derived cell lines is dependent on the type of EBV latency program (type I, II or III)^13^. These observations might explain why while IL-21 has been found to promote apoptosis of DLBCL cell lines via STAT3 activation and c-Myc expression resulting in tumour regression and prolonged survival of mice^16,17^, IL-21 treatment of EBV-positive DLBCL cell lines results in increased cell proliferation^18^. Indeed, when the EBV genome was cleared from these cell lines, a reduction in cell proliferation and an increase in apoptosis was observed upon IL-21 treatment^18^. A large amount of clinical data indicates that IL-21, as a component of the tumour microenvironment, is commonly present in the serum of DLBCL patients and may be involved in the pathogenesis and disease progression of DLBCL^19–22^. In combination with the observation that IL-21 has been used in a variety of clinical trials for the treatment of malignant tumours, it is necessary to further evaluate the pharmacological and physiological effects of IL-21 in the field of non-Hodgkin's lymphoma (NHL) therapy.

A recent report highlights the impressive preclinical antitumor activity and clinical development value of IL-21 in DLBCL but ignores the important impact of EBV in DLBCL^23^. We previously found that IL-21 induced cell proliferation rather than apoptosis on the EBV-positive DLBCL cell line Farage^18^. After obtaining a valuable clinical primary sample of EBV-positive DLBCL, we used RNA-seq to generate a large amount of biological information from a small amount of biological material, overcoming the difficulty of insufficient cell number. This study is an in-depth RNA-seq analysis combining IL-21-treated EBV-positive DLBCL primary cells and the cell line. Furthermore, by comparing the commonalities and differences between EBV-positive and EBV-negative cell lines and the EBV-positive clinical sample, we aimed to identify the mechanism by which IL-21 promotes EBV-positive DLBCL cell proliferation. We predicted a key signalling pathway of IL-21-induced proliferation via expression and activation of cell cycle regulators downstream of *MYC* and AP-1 in EBV-positive DLBCL. Western blotting results of the primary cells and of the EBV-positive DLBCL cell line Farage verified the predictions at the protein expression level that IL-21 specifically upregulated c-Jun, cyclin D2, cyclin E1 expression and Rb phosphorylation. To explore the role of IL-21 in promoting the proliferation of EBV-positive DLBCL cells in vivo, we conducted a complement of experiments and evaluated the in vivo efficacy of IL-21 in EBV-positive DLBCL xenograft tumour experiments.

This work successfully combined dry and wet laboratory research. In the NGS analysis, we not only combined published public data, but also produced valuable, novel NGS data for EBV-positive DLBCL (including data from a rare clinical sample). We expect that this work will contribute to future research on the role of the microenvironment in EBV-positive DLBCL and provide guidance for the proper use of IL-21 in NHL treatment.

## Results

### IL-21 promotes cell viability and survival of primary cells derived from an EBV-positive DLBCL clinical sample

**Figure 1:**
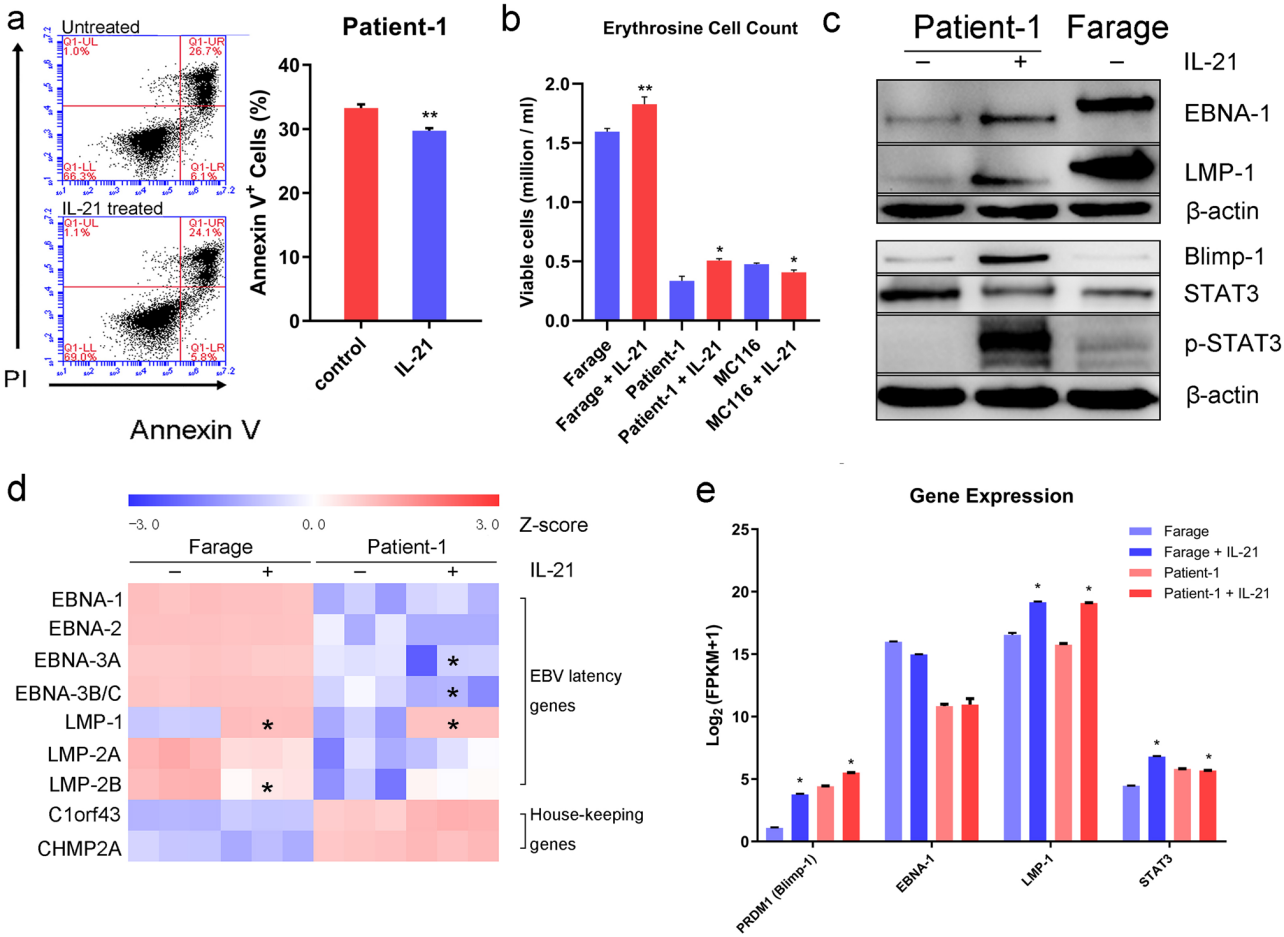
Analysis of apoptosis, viability and gene expression of EBV-positive DLBCL cells after exposure to IL-21. (**a**) The primary cells derived from the EBV-positive DLBCL clinical sample (labelled Patient-1) were treated with IL-21 (100 ng/mL for 48 h) or left untreated. Samples were stained with anti-Annexin V antibodies to measure cell apoptosis by flow cytometry. The experiment was done in triplicate and one representative sample is shown. Statistical analysis of the flow cytometry data was collected from 3 sample replicates. (**b**) The viability of EBV-positive and EBV-negative DLBCL cells was assessed by erythrosine B after 48 h of treatment with IL-21. Data in **a** and **b** are expressed as the means ± SEMs (n = 3); two-tailed t test, **P < 0.01, *P < 0.05. (**c**) The indicated DLBCL cell lysates were probed with the indicated antibodies. β-Actin served as the loading control. (**d**) Z-score normalized log2-(FPKM + 1) of EBV latency genes in gene expression are shown in the heatmap. *C1orf43* and *CHMP2A* were served as the house-keeping gene control. RNA-seq data was collected from 3 sample replicates. (**e**) RNA-seq analysis of selected genes in Farage cells and in the primary cells. Blue indicates decreased expression and red indicates increased expression compared to the IL-21 untreated control. Log_2_-(FPKM + 1) data from the RNA-seq analysis displayed as the means ± SEMs (n = 3). Statistical analysis in **d** and **e** was performed using FDR-based q values; *q < 0.01.

To confirm our previous finding on the EBV-positive DLBCL cell line Farage that IL-21 induced cell proliferation rather than apoptosis, we collected primary cells (named ‘Patient-1’) from a clinical sample of EBV-positive DLBCL. After 48 h of IL-21 treatment, we observed a significant apoptosis reduction in these cells (Fig. 1a) compared to the significantly increased apoptosis in EBV-negative DLBCL primary tumours under similar conditions as previously reported^16^. In addition, IL-21 promoted the viability of the primary cells and of the EBV-positive DLBCL cell line Farage, but reduced viability in the EBV-negative DLBCL cell line MC116 (Fig. 1b). The total cell number of EBV-positive DLBCL cells increased significantly after 48 h with/without IL-21 treatment, indicating cell proliferation in both cases. Using RNA-seq analysis of EBV latency gene transcripts, we found that the EBV-positive DLBCL primary cells expressed the full set of EBV latency genes (indicating a type III latency), which is similar to Farage cells (Fig. 1d). *C1orf43* and *CHMP2A* served as the house-keeping genes^2^. After IL-21 treatment, the expression of Blimp-1 that orchestrates plasma cell differentiation and the viral protein LMP-1 was upregulated in the patient-derived cells as shown by RNA-seq analysis and western blot (Fig. 1c,e), and phosphorylation of STAT3 was also upregulated (Fig. 1e). These results are the same as our previously described in the EBV-positive DLBCL cell line Farage after IL-21 treatment^13,18^. The RNA-seq analysis, combined with our previously reported Western blot results^13,18^ suggests that the expression and regulation of these key genes are similar in Farage cells and the primary cells (Fig. 1c–e), which confirmed our finding in cell lines using a primary sample.

### IL-21 promotes growth and angiogenesis of EBV-positive DLBCL tumours in a NOD/SCID mouse xenograft model.

**Figure 2:**
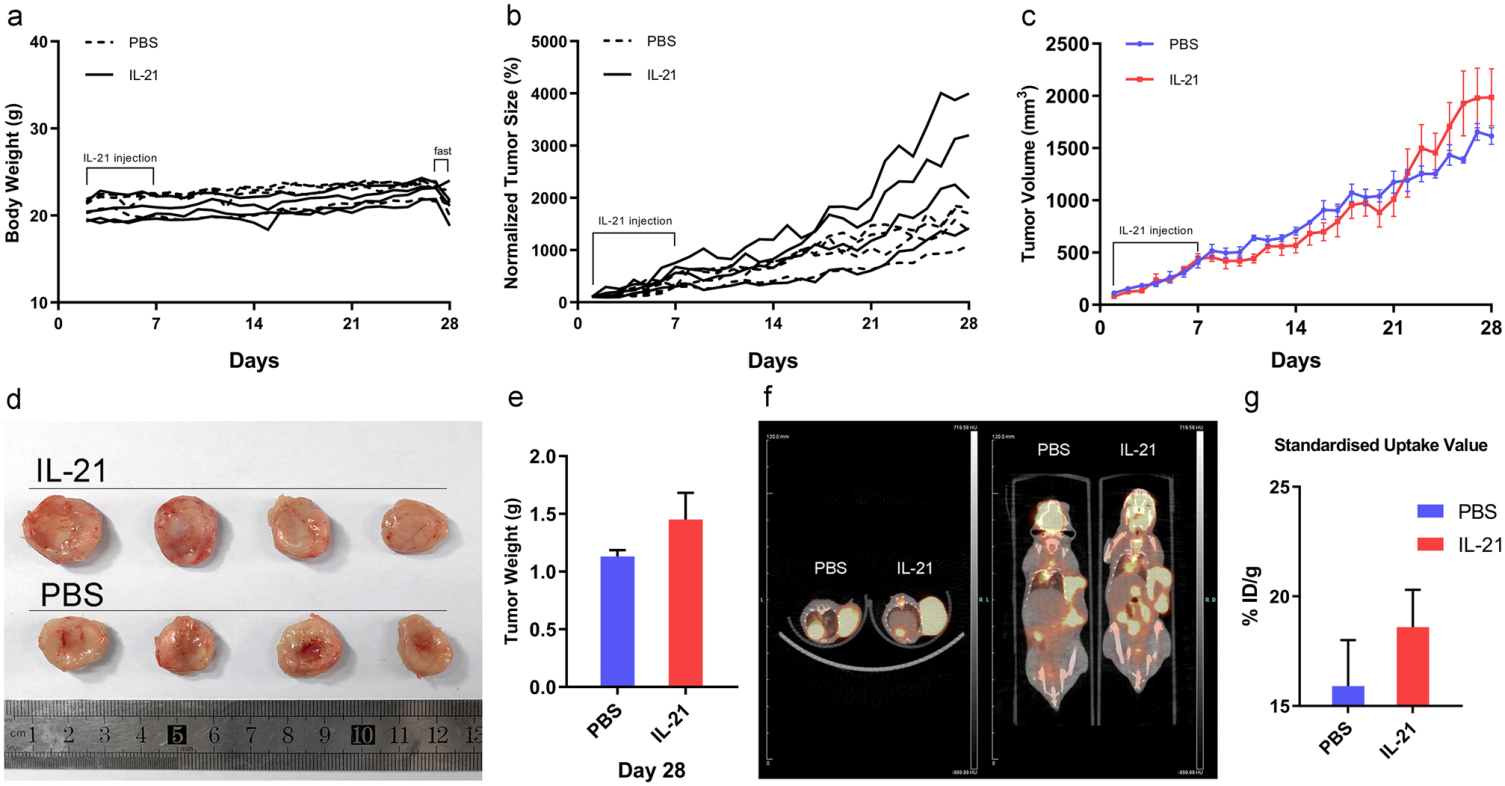
In situ injection of IL-21 promotes the growth of EBV-positive DLBCL tumours in a NOD/SCID mouse xenograft model. (**a**–**c**) Mice (n = 4) bearing subcutaneous xenografts of Farage cells were treated once daily with PBS (control) or IL-21 (10 μg) for 7 consecutive days after the tumour area reached 25 mm^2^. Mouse body weights and tumour volumes were measured daily. Tumour volume of each group is shown as the means ± SEMs (n = 4). (**d**) Tumours isolated from the PBS-treated control and IL-21-treated groups 28 days after tumour implantation. (**e**) Average weight of the tumours were measured 28 days post-implantation. (**f**, **g**) In vivo micro-PET/CT study of ^18^F-FDG uptake. Radioactivity signals were quantified using the standard uptake values. The values in the bar graphs are expressed as the means ± SEMs.

To verify whether IL-21 accelerates EBV-positive DLBCL cell proliferation in vivo, we established a NOD/SCID mouse xenograft model of EBV-positive DLBCL by subcutaneous inoculation of Farage cells and measured tumour growth after IL-21 (10 μg) treatment once daily for 7 consecutive days. Although there was no significant difference in the body weights of tumour-bearing mice (Fig. 2a), tumour proliferation in mice treated with IL-21 was increased (Fig. 2b,c). This was further confirmed by comparison of the size and weights of tumours obtained 28 days post the start of measurement (Fig. 2d,e). To measure tumour areas and evaluate tumour glucose uptake, representative mice were evaluated with ^18^F-FDG micro-PET/CT three weeks after continuous treatment with IL-21 (Fig. 2f,g). A larger signal area was observed in the group treated with IL-21 confirming better tumour growth in these mice. In addition, tumour standard uptake in the IL-21 injection group was increased by 17% compared to that in the control group indicating a higher rate of tumour glucose uptake.

**Figure 3:**
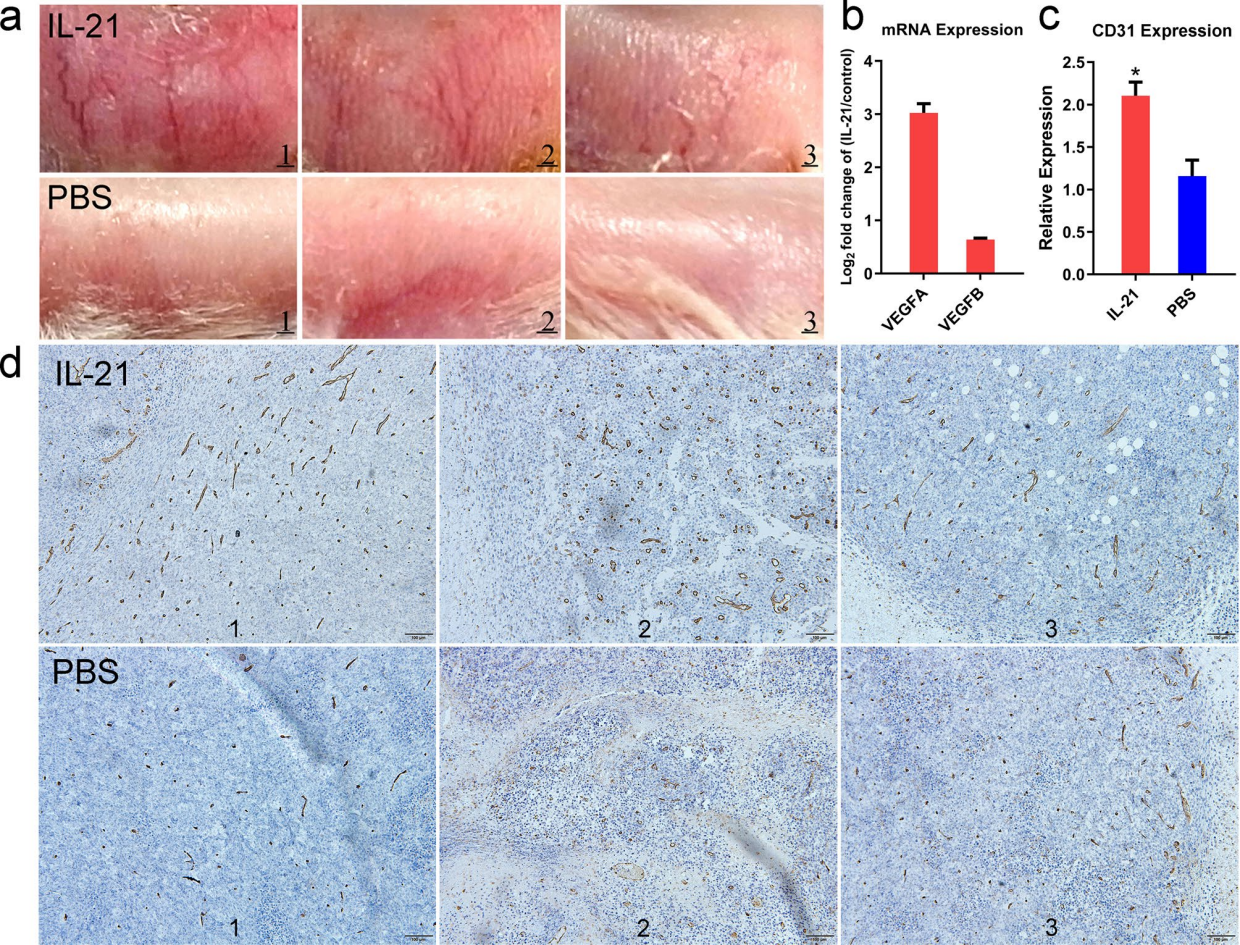
IL-21 treatment promotes angiogenesis in the NOD/SCID mouse xenograft model. (**a**) Epidermal blood vessels observed in the NOD/SCID mouse model. Mice treated with PBS are shown as the control group. Three representative images from different mice are shown for each group. (**b**) Differential expression analysis of the angiogenic genes *VEGFA* and *VEGFB* in Farage 2 days after IL-21/PBS treatment. Data are shown as the means ± SEMs (n = 3). (**c**) Expression of CD31 in tumour tissues from three representative NOD/SCID mice in each group was quantified by ImageJ after staining with anti-CD31 (endothelial marker) antibody. The data represent average protein levels and standard deviations in representative sections. Two-tailed t test, *P < 0.05. (**d**) Immunohistochemical sections of paraffin-embedded tumours from NOD/SCID mice stained with H&E and CD31-specific antibody. Three representative sections for different mice in each group are shown. The scale bar represents 100 μm.

Next, we analyzed the effect of IL-21 treatment on angiogenesis. Consistent with the in vitro gene expression data for *VEGFA* and *VEGFB* showing induction after IL-21 treatment of Farage cells, in vivo we observed promotion of tumour angiogenesis (Fig. 3a,b). After sectioning each of three representative tumours, anti-CD31 (endothelial marker) antibody was used for staining. Sections from the IL-21 group exhibited significantly increased CD31 microvascular staining densities (Fig. 3c,d). These findings may explain the increase in subcutaneous blood vessels around the tumours.

### Gene expression induced by IL-21 exposure is similar in EBV-positive DLBCL primary cells and the Farage cell line

**Figure 4:**
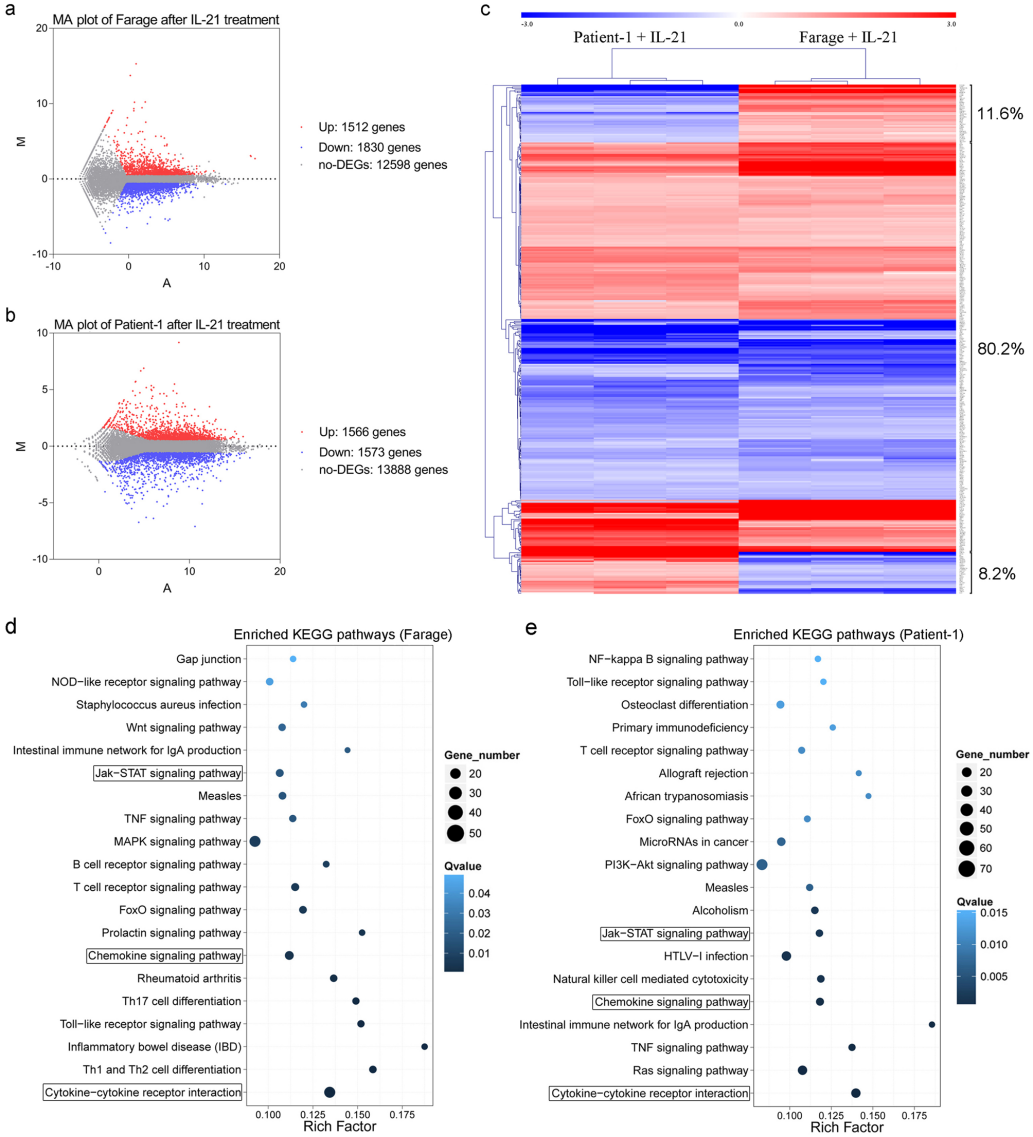
Differentially expression analysis and pathway enrichment analysis of genes in EBV-positive DLBCL cells treated with IL-21. (**a**, **b**) EBV-positive DLBCL primary cells and Farage cells were treated with IL-21 (100 ng/mL for 48 h) or left untreated and gene expression was analysed by RNA-seq. MA plots show the displayed A values (average log2 transformed expression value) and M values (fold change in the log2 transformed expression value) of the data from Farage cells and the primary cells. The red dots indicate differentially expressed genes (DEGs) that were upregulated (fold-change > 1.5, q values < 0.01). The blue dots indicate DEGs that were downregulated (fold-change < − 1.5, q values < 0.01). The grey dots represent genes that were not differentially expressed. (**c**) Unsupervised hierarchical clustering of expression fold change data for differentially expressed disease-associated genes (fold-change > 1.5 or < − 1.5, q values < 0.01) in the primary and Farage cells. Each group is comprised of three pooled biological replicates. (**d**,** e**) Top-ranked pathways enriched in the differentially expressed genes (DEGs) in KEGG pathway analysis. The colours indicate the q-values (high: white, low: blue). Lower q-values indicate more significant enrichment. The dot size reflects the number of DEGs (larger dots indicate a larger number). ”Rich Factor” indicates the value of the enrichment factor, which is the quotient of the foreground value (the number of DEGs) and the background value (the total number of genes). The larger the value, the more significant is the enrichment. Boxed text indicates common top-ranked pathways in the primary cells and Farage cells.

The previous results suggested that IL-21 treatment of EBV-positive DLBCL primary cells and cells from the Farage cell line had similar effects. To study whether this reflected similar gene expression regulation in the two types of cells following IL-21 exposure, we analysed the global gene expression of the two types of cells before and after 48 h of IL-21 exposure. The MA plot shows that IL-21 treatment upregulated the expression of 1512 and 1566 genes in Farage and primary cells, respectively, and downregulated the expression of 1,830 and 1,573 genes (Fig. 4a,b). To identify the lymphoma associated genes, we performed a real-time literature analysis using PALM-IST (Pathway Assembly from Literature Mining—an Information Search Tool)^24^. We scanned 61,835 article abstracts in the PubMed databases using lymphoma as the main keyword and generated a disease-associated gene (DAG) library (Supplementary Table S1). Based on the DAG library, we performed hierarchical clustering analysis of the fold change of each differential expressed gene (DEG) for each independent sample in the Farage and primary cells groups. The results indicated that IL-21 showed similar regulation of DAGs in Farage cells and the primary cells. Specifically, 80.2% of DAGs in the Farage and primary cells had similar regulatory trends after IL-21 treatment (Fig. 4c).

To identify the most significant signalling pathways regulated by IL-21 in EBV-positive DLBCL, phyper was used to perform KEGG pathway functional enrichment analysis for all DEGs. After IL-21 treatment, DEGs in the Farage group and the primary cells group were highly enriched in the cytokine-cytokine receptor interaction pathway, chemokine signalling pathway and JAK-STAT signalling pathway (Fig. 4d,e). In addition, DEGs enriched in the JAK-STAT signalling pathway showed overall upregulation (Supplementary Fig. S2).

### The master regulator of cell proliferation MYC is upregulated in EBV-positive DLBCL primary and Farage cells upon IL-21 exposure

**Figure 5:**
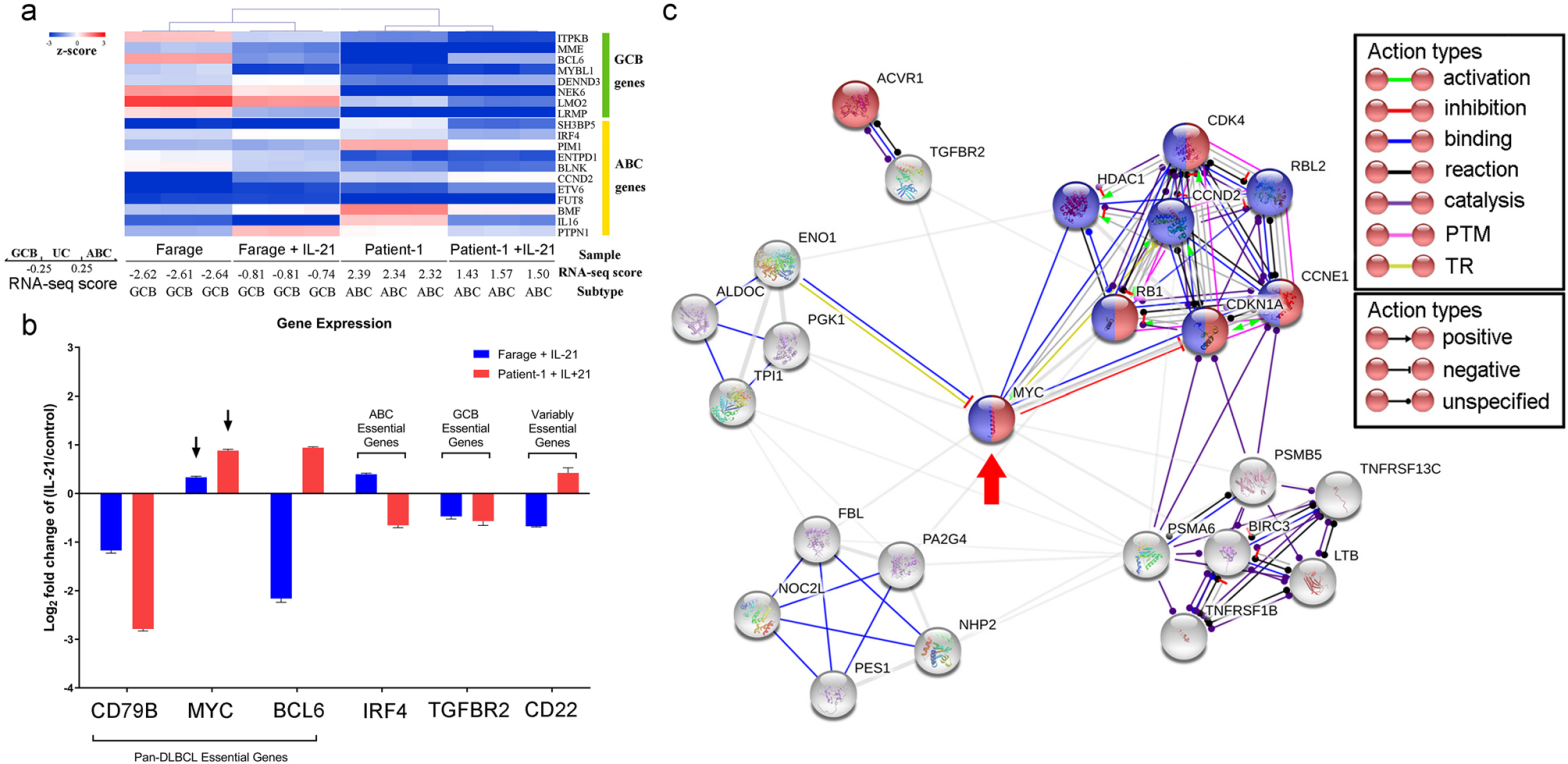
*MYC* plays a key role in IL-21-induced proliferation in Farage cells and the primary cells. (**a**) An RNA-seq gene expression classifier distinguishes germinal centre B cell-like (GCB) DLBCL, unclassified (UC) DLBCL and activated B cell-like (ABC) DLBCL. The ABC- and GCB-specific scores were computed for each sample by averaging the z-scores for ABC and GCB genes, respectively. (**b**) Gene expression profile of DLBCL genetic drivers in EBV-positive DLBCL after IL-21 treatment. Statistical analysis was performed using FDR-based q values, and all data shown were significant with a q value of < 0.01. The arrow indicates a common upregulation of *MYC* in both groups after IL-21 treatment. (**c**) The MCODE plugin of Cytoscape was used to extract subnetworks in STRING-based network analysis for co-DEGs in Farage cells and primary cells. Cluster 3 was generated from the entire picture, and the core module was extracted from cluster 3 by disabling the Fluff function in MCODE. The nodes in blue indicate genes involved in cell cycle regulation (GO: 0051726, FDR < 0.0001), and the nodes in red indicate genes involved in the G1/S transition in the mitotic cell cycle (GO: 000082, FDR < 0.0001). The arrow indicates a central position of *MYC* in the core module. PTM, posttranlational modification; TR, translational regulation.

To identify the main effector of IL-21-induced EBV-positive DLBCL cell proliferation, we applied an RNA-sequencing-based ABC/GCB calling method combining corresponding CRISPR screening data to seek more hints^25^. This method classifies DLBCL tumours based on RNA-seq gene expression data into activated B cell-like (ABC) and germinal centre B cell-like (GCB) types. Patients with ABC-type DLBCL have a lower overall survival rate than patients with GCB-type DLBCL. In our study, based on the RNA-seq data characteristics, Farage cells were classified as GCB DLBCL, and the primary cells were classified as ABC DLBCL (Fig. 5a). The calling results remained unchanged in both groups after IL-21 treatment. Besides, six DLBCL driver genes^25^, namely *CD97B*, *MYC*, *BCL6*, *IRF4*, *TGFBR2* and *CD22*, were differentially expressed in both types of cells upon IL-21 exposure (Fig. 5b). Notably, *MYC*, a pan-DLBCL (among all DLBCL subtypes) essential gene for cell survival, was significantly upregulated by IL-21 in both Farage and primary cells.

### Molecular interaction network analysis identifies a cell cycle module in which MYC plays a central role

To identify the molecular network involved in IL-21-induced cell proliferation, we conducted STRING network analysis using functional differential expression data from Farage cells and the primary cells. We obtained a molecular interaction network containing 350 genes and 683 edges (Supplementary Fig. S3e, Supplementary Table S2). To analyse the submodules, we used the MCODE plugin (v1.4.2) of Cytoscape (v3.5.1) to extract subnetworks from the entire molecular interaction network (Supplementary Fig. S3b–d), with a cut-off for the node connectivity degree of greater than 3. Based on the results of functional enrichment analysis (Supplementary Table S3–S6), we found that cluster 1 (Supplementary Fig. S3d, Supplementary Table S4) and cluster 2 (Supplementary Fig. S3c, Supplementary Table S5) are mainly involved in infection and immune regulation, and that cluster 3 (Supplementary Fig. S3b, Supplementary Table S6) is mainly involved in cell death and the cell cycle. To obtain the core molecular interaction module for IL-21-induced cell proliferation, we extracted a subnetwork consisting of 25 genes and 73 edges from cluster 3 by disabling the Fluff function in MCODE (Fig. 5c). The genes that form this core module are significantly enriched in the biological processes of cell cycle regulation and mitotic cell cycle G1/S transition and significantly enriched in the KEGG cell cycle pathway (Table 1, Supplementary Table S7). This subnetwork module describes the regulatory effect of IL-21 on cell cycle progression in EBV-positive DLBCL from a molecular interaction perspective. We found from this subnetwork module that *MYC* occupies a central position in the module with regard to cell cycle regulation.

**Table 1:** Highly enriched biological processes (gene ontology, GO) and KEGG pathways in the core module from the whole network.

Pathway ID	Pathway description	Gene count		False discovery rate
**Biological process (GO)**				
GO:0051726	Regulation of cell cycle		12	1.53E−06
GO:0000082	G1/S transition of mitotic cell cycle		7	3.39E−06
GO:0051247	Positive regulation of protein metabolic process		13	3.39E−06
GO:0051246	Regulation of protein metabolic process		15	8.91E−06
GO:0007049	Cell cycle		12	1.12E−05
**KEGG pathway**				
hsa04110	Cell cycle		8	3.38E−10
hsa05220	Chronic myeloid leukaemia		6	2.85E−08
hsa05203	Viral carcinogenesis		7	1.52E−07
hsa05200	Pathways in cancer		8	2.02E−07
hsa05166	HTLV-I infection		7	8.56E−07

### IL-21 promotes cyclins in G1/S phase transition in EBV-positive DLBCL but not in EBV-negative DLBCL

**Figure 6:**
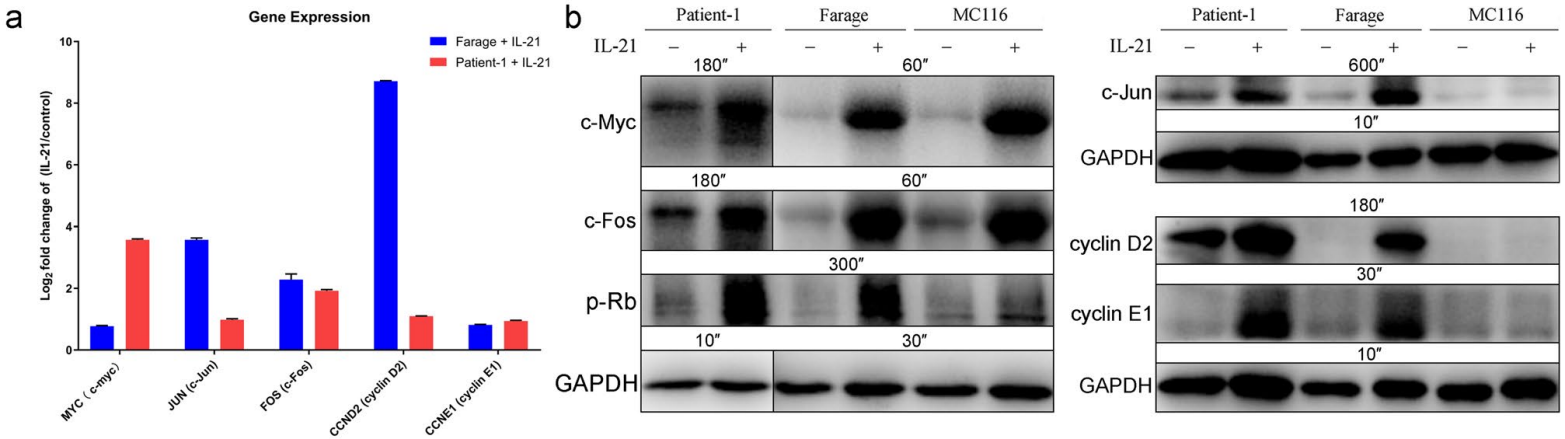
IL-21 promotes the expression of markers of the G1/S phase transition in EBV-positive DLBCL but not in EBV-negative DLBCL cells. (**a**) Differential gene expression analysis of the RNA-seq data of key genes in the core molecular module in Farage cells and primary cells treated with IL-21 (100 ng/mL for 48 h) compared with expression in untreated cells.. The log2 fold changes in gene expression are shown as the means ± SEMs (n = 3). Statistical analysis was performed using FDR-based q values, and all data shown were significant with a q value of < 0.01. (**b**) The indicated DLBCL cell lysates were probed with the indicated antibodies. GAPDH was used as the loading control. MC116 cell line was used as an EBV-negative DLBCL comparison group. The exposure time for each protein has been indicated accordingly.

**Figure 7:**
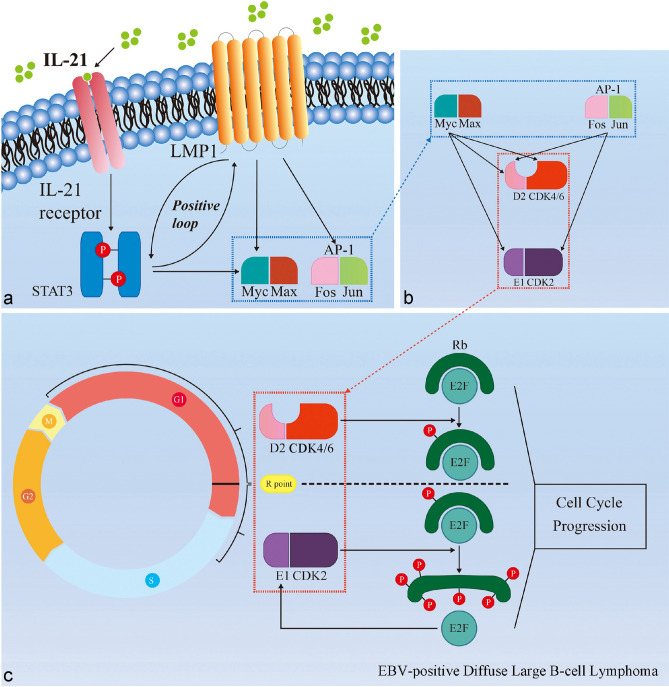
Hypothetical working model of the promoted G1/S phase transition by IL-21 in EBV-positive DLBCL cells. A working model is proposed where IL-21 stimulation enhances STAT3 phosphorylation, subsequently stimulating LMP-1 expression, and establishing a positive feedback loop, resulting in upregulation of c-Myc and AP-1 expression. c-Myc and AP-1 promote restriction point transition via upregulation of cyclin D2 and cyclin E1, which promotes hyperphosphorylation of Rb, thereby promoting cell cycle progression.

To confirm the results from RNA-seq and the network analysis, we extracted the differential expression results from the RNA-seq data and used western blotting to evaluate changes in gene expression at the protein level (Fig. 6a,b). We found that consistent with its effect in Farage cells, IL-21 induced upregulation of c-Myc, c-Jun, c-Fos, cyclin D2 and cyclin E1 protein expression in the primary cells (Fig. 6b). Subtype-specific DEG analysis of cyclin D and E confirmed that only cyclin D2 and cyclin E1 are significantly regulated by IL-21 in both primary cells and Farage (Supplementary Fig. S4). Since these cyclins are highly correlated with the restriction (R) point transition in the cell cycle, we further examined the phosphorylation status of Rb, which is a negative regulator of cell division whose phosphorylation is associated with cell cycle entry. After IL-21 treatment, we observed increased Rb phosphorylation in both Farage cells and the primary cells (Fig. 6b). The relative protein levels were similar to the differential expression results from RNA-seq analysis (Fig. 6a,b). By contrast, in the EBV-negative DLBCL cell line MC116, IL-21 did not induce upregulation of c-Jun, cyclin D2, and cyclin E1 (Fig. 6b) and no clear increase in the levels of phosphorylated Rb protein was detected in MC116 cells (Fig. 6b). Therefore, we propose a hypothetical model for growth regulation by IL-21 in EBV-positive DLBCL in which IL-21 signalling leads to STAT3 phosphorylation and induces LMP-1 expression to form a positive autoregulatory loop, which amplifies the signals for Myc and AP-1 (composed of homoor heterodimers formed by related Jun and Fos family proteins) transcription (Fig. 7a). The activation of c-Myc and AP-1 results in increased cyclin D2 and cyclin E1 expression (Fig. 7b), promotes R point transition, and ultimately leads to hyperphosphorylation of Rb which accelerates cell cycle progression (Fig. 7c).

## Discussion

In the present work, we confirmed previous observations of IL-21-induced proliferation in EBV-positive DLBCL^18^ using an EBV-positive primary clinical sample and an in vivo NOD/SCID mouse xenograft model. These results differ from the tumour regression observed in a previous mouse study using MC116, an EBV-negative DLBCL^16^.

By RNA-seq analysis using hierarchical clustering, we found that the direction of the regulation of DEGs in the primary cells and in Farage cells after IL-21 treatment was similar. This similarity suggests that Farage cells are an appropriate research tool for the study of type III latency EBV-positive DLBCL. We also conducted a joint analysis of the primary cells and Farage cells with reference to the DLBCL subtype calling model^25^ and corresponding CRISPR screening data proposed by the Dave laboratory^25,26^. Here, genes associated with ABC/GCB classification were used to classify samples into subgroups, and an RNAseqSubtypeScore^25^ was applied accordingly. This score indicates the difference of calculated characteristic value between ABC and GCB. Our results showed that the unsupervised hierarchical clustering did not distinguish the IL-21 treated cells and IL-21 untreated cells (Fig. 5a). Interestingly, according to the ABC/GCB classification based CRISPR oncogene targets^25^, our results showed that *MYC* is the only DLBCL essential gene that was upregulated by IL-21 in both the primary cells and Farage cells (Fig. 5a,b). Based on the role of *MYC* in cell proliferation, we hypothesised that *MYC* plays a key role in IL-21-induced proliferation of EBV-positive DLBCL cells. Using bioinformatic methods, we extracted the core subnetworks of co-DEGs in Farage cells and the primary cells. Functional enrichment analysis revealed a subnetwork involved in the regulation of the mitotic cell cycle (Table 1), with *MYC* at the centre of the core module (Fig. 5c).

Myc acts as the primary transcriptional centre for multiple networks in B cells^27,28^. It is a known target of the STAT3 transcription factor^29–31^ and previous publications have identified several target genes of Myc^32–34^. Myc directly and indirectly activates the expression of cyclin D2 and cyclin E to promote R point transition^32,35–39^. The level of D-type cyclins is primarily controlled by extracellular signals^40^, which, together with CDK4/6, are responsible for inducing Rb phosphorylation. Once cells move through the R point, the remaining cyclins, including E-type cyclins, behave in a pre-programmed manner until the end of M phase and no longer respond to extracellular signals. It is worth noting that although IL-21 caused upregulation of c-Myc in MC116 cells, it did not cause upregulation of c-Jun, cyclin D2 or cyclin E1, and no substantial increase in the levels of phosphorylated Rb protein was observed upon IL-21 treatment. This pattern suggests a significant difference in the role of IL-21 in regulating proliferation in EBV-positive DLBCL and EBV-negative DLBCL.

The effect of IL-21 on the fate of B cells depends on their activation state and developmental stage. In some haematological malignancies, such as multiple myeloma, Hodgkin's lymphoma and Burkitt lymphoma, IL-21 can induce the proliferation of neoplastic B cells^23^. Although IL-21 has no mitogenic effect on isolated normal human B cells, it significantly induces the proliferation of B cells co-stimulated with anti-CD40 monoclonal antibodies^41^. Given the evidence that LMP-1, an oncoprotein encoded by EBV, mimics the constitutively activated CD40 signal in B lymphocyte responses^42^, it is plausible that IL-21 induces EBV-positive DLBCL cell proliferation by a similar mechanism. It has also been reported that cells with high LMP-1 expression are responsible for the poor drug response of EBV-positive DLBCL^43^. Combining these results with our previous observations, we hypothesise that the LMP-1 induced in EBV-positive DLBCL cells may be a key factor for proliferation.

LMP-1 leads to activation of the JAK/STAT signalling pathway^44^ and is critical for the proliferation and survival of EBV-positive B cells^13,18^. Evidence suggests that activated STAT3 acts as an activator of the LMP-1 L1-TR promoter^45^, which creates a positive autoregulatory loop since STAT3 itself can be activated by LMP-1^44^. Our results showed a significant increase in STAT3 phosphorylation as well as LMP-1 expression after IL-21 treatment in both Farage cells and the patient-derived primary cells. LMP-1 signalling has been shown to have an important effect on the G1/S transition in human B cells^46^ with LMP-1 specifically inducing mitogenic B cell activation via Myc upregulation^47^.

Importantly, LMP-1 also induces mitogenic B cell activation via members of the Jun/AP-1 family^47^. After IL-21 treatment, the mRNA and protein levels of c-Jun and c-Fos were significantly increased in the EBV-positive DLBCL cell line and the primary cells. It is known that the transcription of Jun and other AP-1 family members is rapidly and transiently stimulated by many external signals that promote mitogen-induced cell cycle progression^38,48^. Additionally, there is evidence that AP-1 blockade inhibits Rb phosphorylation, decreases the expression of major cyclins in the G1 phase of the cell cycle (D and E cyclins), and decreases the activity of CDK2 and CDK4^37^. Collectively, these indications suggest that the different outcomes of IL-21 signalling in EBV-negative DLBCL and EBV-positive DLBCL cells, both showing an upregulation of *MYC*, are most likely due to the presence/absence of LMP-1.

Moreover, we demonstrated that the expression of CCL22 was significantly decreased and that the expression of CXCL10 was considerably increased in both Farage cells and the primary cells after IL-21 treatment (Supplementary Fig. S5). Low CCL22 expression and the presence of EBV are associated with decreased survival in patients with DLBCL^49^. High expression of CXCL10 is closely associated with poor survival in patients with DLBCL and secondary central nervous system involvement was more frequent in the high CXCL10 group^50,51^. Therefore, in the EBV-positive DLBCL cell line and the primary cells, upregulation of CXCL10 and downregulation of CCL22 by IL-21 might be detrimental to DLBCL prognosis.

The lack of sufficient cell numbers of clinical samples that can be used for functional studies is one of the major reasons hindering the investigation of EBV-positive DLBCL. This problem can be effectively solved by NGS technology, which produces large amounts of bioinformatic data from a limited amount of material. In our literature analysis in the PubMed database, we used lymphoma as the keyword to extract lists of hotspot genes and build disease-related gene sets to successfully expand our knowledge of disease-related transcriptional regulatory signals. After identifying the core transcription subnetwork through network analysis and enrichment analysis, we validated the prediction results using wet laboratory methods. Our comprehensive research findings show that NGS can satisfactorily match these research protocols and help to predict core molecular mechanisms.

Our study shows that the inappropriate use of IL-21 in patients with EBV-positive DLBCL not only may fail to achieve therapeutic effects but also may lead to accelerated proliferation of DLBCL. In addition, this work suggests that IL-21, as a component of the tumour microenvironment, may also contribute to the proliferation of EBV-positive DLBCL cells. In drug development, key regulators such as the IL-21 receptor and LMP-1 may be valuable as therapeutic targets for EBV-positive DLBCL, and monoclonal antibodies or small molecule antagonists targeting these regulators may be beneficial for the survival of patients. Further functional studies of the key regulatory nodes identified in the current study are needed to confirm our computational data. Although it is currently difficult to integrate large sample resources into systematic research, we hope that this analysis on a clinical sample can further increase researchers' attention to EBV status in DLBCL treatment and supplement biological information in this field.

## Materials and methods

### Reagents, antibodies, cell lines, and cytokine treatments of cell lines

The EBV-positive diffuse large B cell lymphoma (DLBCL) cell line Farage was kindly provided by Dr. Hannah Ben-Bassat (Laboratory of Experimental Surgery, Hadassah University Hospital, Jerusalem)^52^. Farage and MC116 (EBV-negative DLBCL cell line) were cultured in RPMI medium supplemented with 10% foetal bovine serum, 50 units/mL penicillin and 50 g/mL streptomycin. The EBV-positive DLBCL primary cells Patient-1, generated from ascites, was maintained in RPMI medium supplemented with 20% foetal bovine serum, 50 units/mL penicillin and 50 g/mL streptomycin. Recombinant human IL-21 was purchased from Peprotech.

### Isolation of primary EBV-positive DLBCL B lymphocytes

All procedures followed were in accordance with the ethical standards and approved by the responsible committee of the First Affiliated Hospital of Nanjing Medical University, Jiangsu Province Hospital (2017-SRFA-167). Primary cells were derived from ascites of the patient and informed consent was obtained from all subjects. Density gradient separation of primary cells was performed with lymphocyte separation medium (Sinopharm Chemical Reagent Co., Ltd.). B lymphocytes were further isolated via negative selection using a BD IMag Human B Lymphocyte Enrichment Set—DM according to the manufacturer’s instructions. In brief, the biotinylated Human B Lymphocyte Enrichment Cocktail (component 51-9003033) consists of the following biotinylated antibodies: anti-human CD3 (clone UCHT1), anti-human CD41a (clone HIP8), anti-human CD43 (clone L60), and anti-human CD235a (clone GA-R2). These monoclonal antibodies recognise antigens expressed on erythrocytes, platelets, and peripheral leukocytes, but not on B lymphocytes. The BD IMag Streptavidin Particles Plus–DM (component 51-9000810) magnetic particles, which contain streptavidin covalently conjugated to their surfaces, were used for the B lymphocyte isolation.

### Flow cytometry

An Annexin V-FITC/PI Apoptosis Kit from Invitrogen (Waltham, MA, USA) was used to detect apoptosis by flow cytometry (BD Accuri C6) according to the manufacturer’s instructions.

### Cell viability assay

The viability of cells was estimated using an Erythrosin B (Sigma-Aldrich, St. Louis, MO, USA) exclusion assay analysis of cultures treated with IL-21 (100 ng/mL) for 48 h. Farage and MC116 were cultured at an initial density of 6 × 10^4^ cells/mL when treated, and the primary cells Patient-1 were cultured at an initial density of 3 × 10^5^ cells/mL.

### NOD/SCID mouse model

A murine EBV-positive DLBCL xenograft model was used to assess the in vivo effect of IL-21. Six-week-old female NOD/SCID mice weighing 16–20 g (Beijing Vital River Laboratory Animal Technology Co., Ltd., Animal Certificate: SCXK Jing 2016-0006) were subcutaneously inoculated with Farage cells (1 × 10^7^ cells/200 μL, PBS:Matrigel = 1:1). The care of mice was in accordance with institution guidelines and they were monitored daily for tumour growth. When the tumour area reached 25 mm^2^, mice were treated intratumorally once daily with IL-21 (10 μg) or PBS for 7 consecutive days following a previously described protocol^16^. Tumours were measured with standard callipers using the two largest perpendicular axes. Tumour-bearing mice were assessed for weight loss and tumour size daily.

### Micro-PET/CT imaging

Fasting NOD/SCID mice bearing xenografts were subjected to ^18^F-FDG micro-PET/CT analysis performed with an Inveon Multimodality system (Siemens, Munich, Germany). Mice were anaesthetised with isoflurane prior to a single injection of 3.7 MBq ^18^F-FDG via the tail vein. Forty minutes after administration of the tracer, mice were placed on the PET scanner bed and maintained under continuous anaesthesia during the study. The standardised uptake value (SUV) was calculated as follows: decay-corrected activity (kBq) per mL of tissue volume/injected ^18^F-FDG activity (kBq) per g of body weight^53^.

### Immunohistochemistry

Tumour tissues were fixed in 4% paraformaldehyde, embedded in paraffin, and sectioned before staining with haematoxylin–eosin (H&E) and antibodies against CD31 (GB11063-2, Servicebio). The areas of positive staining were quantified using ImageJ software and expressed as a percentage.

### RNA sequencing analysis

The RNA-seq process was performed on the BGISEQ-500 platform (The Beijing Genomics Institute). Sequencing reads containing low-quality reads, adaptor-polluted reads and reads with a high content of unknown bases were removed by SOAPnuke (v1.5.2) before downstream analyses. Clean reads were mapped to reference transcripts using Bowtie2 (v2.2.5)^54^. Gene expression was calculated for each sample using RSEM (v1.2.12)^55^. The DEGseq algorithm was used to identify differentially expressed genes (DEGs)^56^. Gene Ontology (GO) classification, functional enrichment analysis, KEGG pathway classification^57–59^ and functional enrichment analysis were performed for the DEGs.

### Network construction and analysis

The molecular interaction information was obtained from the STRING 11.0 online database (https://string-db.org)^60^. Interactions with a confidence score of greater than 0.400 were selected for network construction. The Molecular Complex Detection (MCODE) plugin^61^ in Cytoscape software (v3.5.1) was used to further divide the whole network into modules using a cut-off for the node connectivity degree of greater than 3 (Haircut: enabled; Fluff: enabled; Node Score Cut-off: 0.2; K-Core: 2; Max. Depth: 100), generating Clusters 1–3 (Supplementary Fig. S3). By disabling the Fluff function, which expands the core cluster by one neighbour shell, we further extracted the core module through Cluster 3 based on the above parameters.

### Western blot analysis

Cells were lysed in RIPA lysis buffer following 2 days of IL-21 treatment. Protein samples were separated by SDS-PAGE under reducing conditions and were transferred to a methanol-activated PVDF membrane. After blocking the membranes for 2 h at room temperature with 5% nonfat dried milk, membranes were incubated with primary antibody overnight at 4 °C. After incubation with secondary antibody for 2 h at room temperature, immunoreactive bands were visualized by Immobilon Western chemiluminescent HRP substrate (Millipore, Darmstadt, Germany)^62^. The following antibodies were used as primary antibodies in immunoblotting: AC-15 (mouse anti-human β-actin; Sigma-Aldrich), 3H2-E8 (mouse anti-Epstein-Barr Virus Blimp-1, Novus); 0211 (mouse anti-Epstein-Barr Virus EBNA-1, Santa Cruz), CS.1–4 (mouse anti-Epstein-Barr Virus LMP; DAKO), D84C12 (rabbit anti-c-Myc), 124H6 (mouse anti-human Stat3), M9C6 (mouse anti-human Phospho-Stat3 (Tyr705)), 9F6 (rabbit anti-human c-Fos), 60A8 (rabbit anti-human c-Jun), D52F9 (rabbit anti-human Cyclin D2), HE12 (mouse anti-human Cyclin E1), 4H1 (mouse anti-human Rb), and 9308 (rabbit anti-human Phospho-Rb (Ser807/811)) from Cell Signalling Technology.

### Statistical analysis

All statistical analyses, excluding those for RNA-seq, were performed using GraphPad Prism 7. Differences between individual groups were tested for statistical significance using unpaired two-tailed Student’s t tests (*P < 0.05; **P < 0.01; ***P < 0.001; ****P < 0.0001.). The false discovery rate (FDR) was used in functional enrichment analyses; the q value was utilised as an FDR-based measure of significance for differential expression analysis^63^.

## Supplementary information

Supplementary Figures.

Supplementary Table 1.

Supplementary Table 2.

Supplementary Table 3.

Supplementary Table 4.

Supplementary Table 5.

Supplementary Table 6.

Supplementary Table 7.

## Data Availability

The datasets used and/or analyzed during the current study are available from the corresponding author on reasonable request.
