# Supplementary material for: IL-21 Stimulates the expression and activation of cell cycle regulators and promotes cell proliferation in EBV-positive diffuse large B cell lymphoma
Source: Sci Rep. 2020 Jul 23;10:12326. doi: 10.1038/s41598-020-69227-0 (PMC7378064; doi:10.1038/s41598-020-69227-0)
Supplement: Supplementary file 1 — Supplementary Figures. [file 41598_2020_69227_MOESM1_ESM.docx]

**Title**: IL-21 Stimulates the Expression and Activation of Cell Cycle Regulators and Promotes Cell Proliferation in EBV-positive Diffuse Large B Cell Lymphoma

**Authors:** Yuxuan Wang^1^, Chengcheng Wang^1^, Xiyunyi Cai^1^, Chang Mou^1^, Xueting Cui^1^, Yingying Zhang^1^, Feng Ge^1^, Hao Dong^1^, Yuanyuan Hao^1^, Lei Cai^1^, Shuting Wu^1^, Chenjie Feng^1^, Jiamin Chen^1^, Jianyong Li^2^, Wei Xu^2^, Lei Fan^2,#^, Weijia Xie^3,4^, Yue Tong^3,5,6^, Harvest Feng Gu^7^, Liang Wu^1,#^

^1^Jiangsu Key Laboratory of Drug Screening, China Pharmaceutical University, Nanjing, China.

^2^Department of Hematology, The First Affiliated Hospital of Nanjing Medical University, Jiangsu Province Hospital, Collaborative Innovation Center for Cancer Personalized Medicine, Nanjing, China.

^3^State Key Laboratory of Natural Medicines, China Pharmaceutical University, Nanjing, China.

^4^Department of Medicinal Chemistry, China Pharmaceutical University, Nanjing, China.

^5^Jiangsu Key Laboratory of Druggability of Biopharmaceuticals, China Pharmaceutical University, Nanjing, China.

^6^School of Life Science and Technology, China Pharmaceutical University, Nanjing, China.

^7^School of Basic Medicine and Clinical Pharmacy, China Pharmaceutical University, Nanjing, China.

**Supplementary Figures**

**
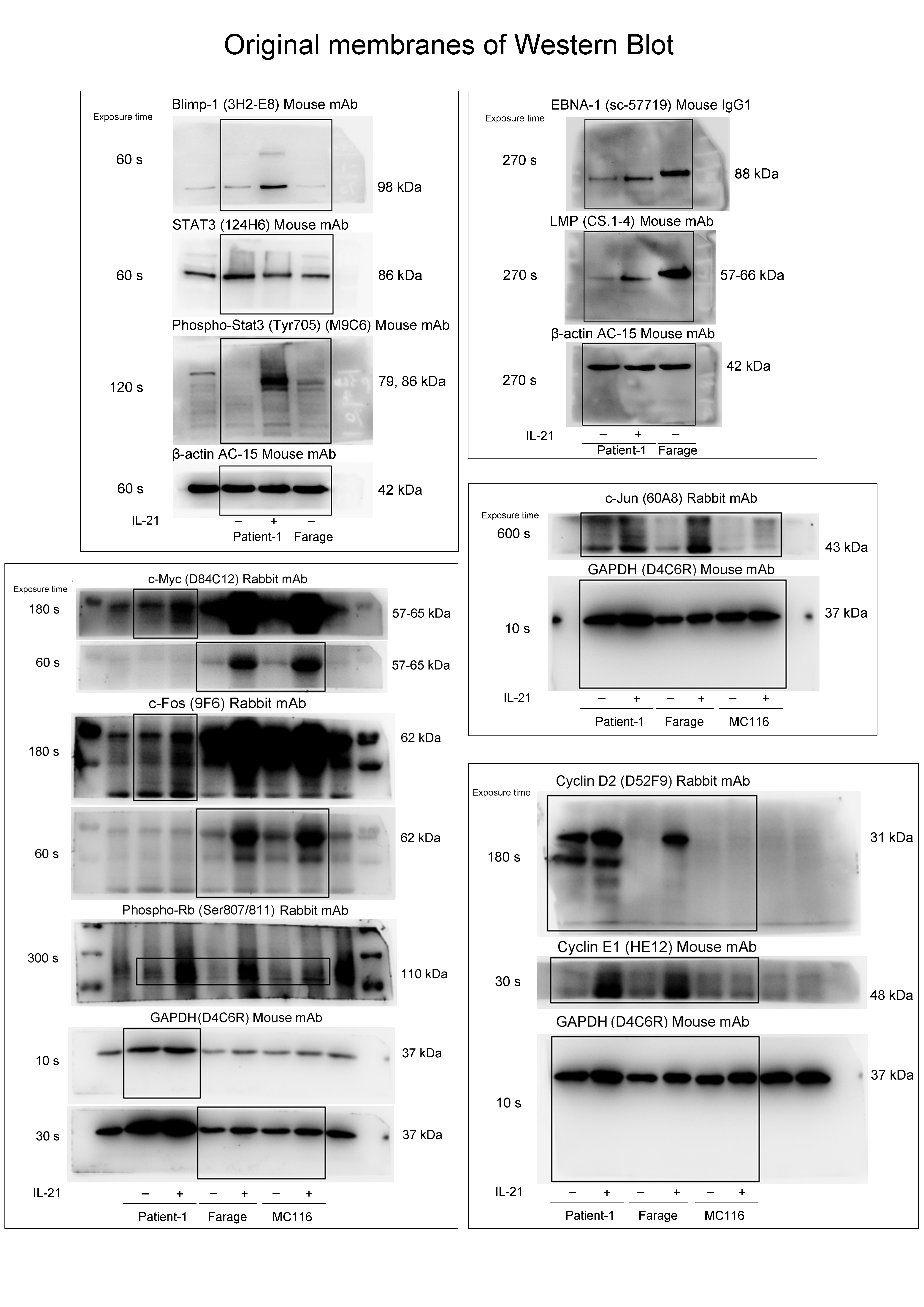
**

**
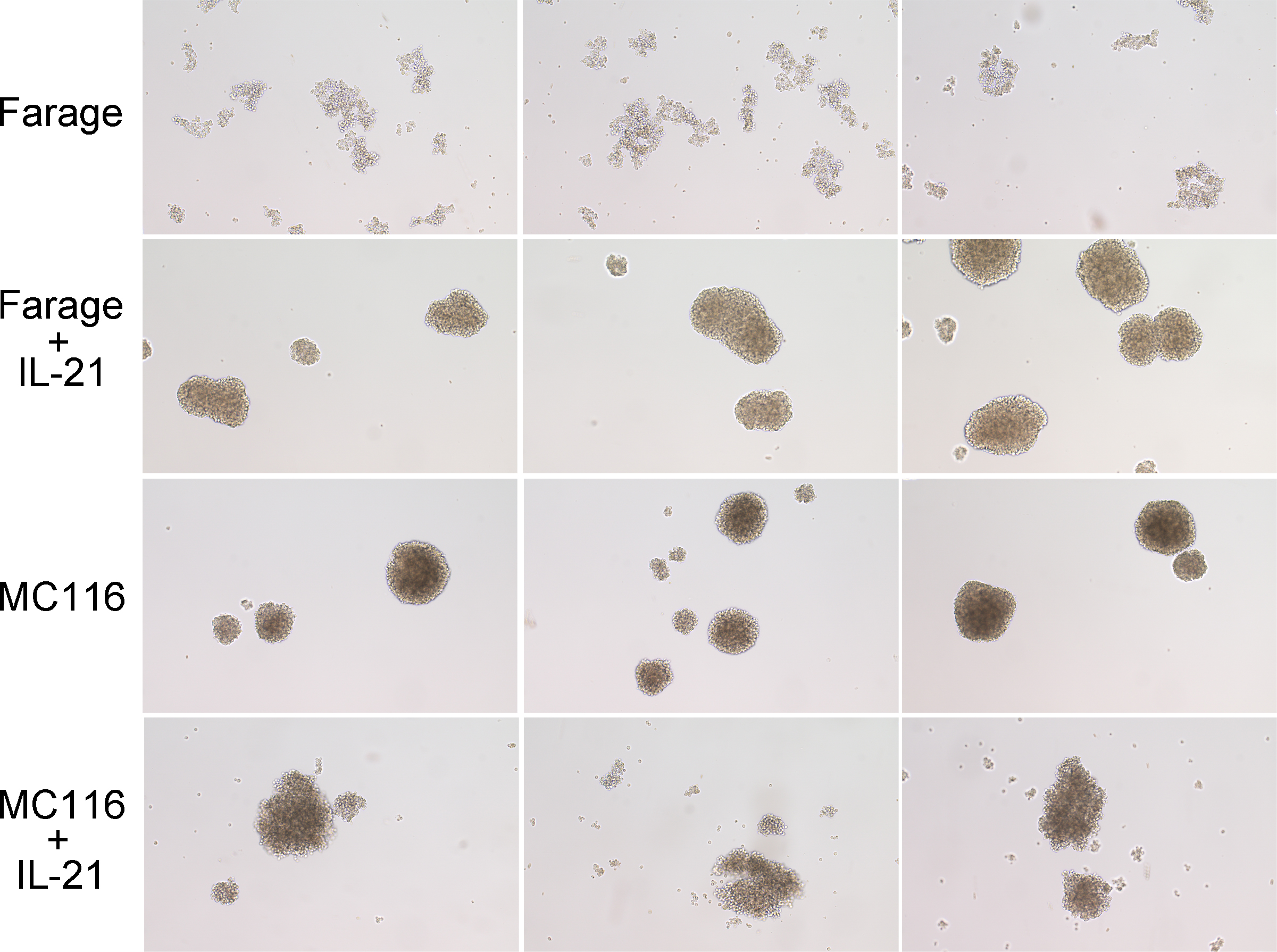
**

**Figure S1.** Cell morphology of DLBCL cell lines after IL-21 treatment. EBV-positive DLBCL cells Farage and EBV-negative DLBCL cells MC116 were treated with IL-21 (100 ng/ml for 48 h) or left untreated. The experiment was done in triplicate and three representative images from different culture flasks were shown for each group.

**
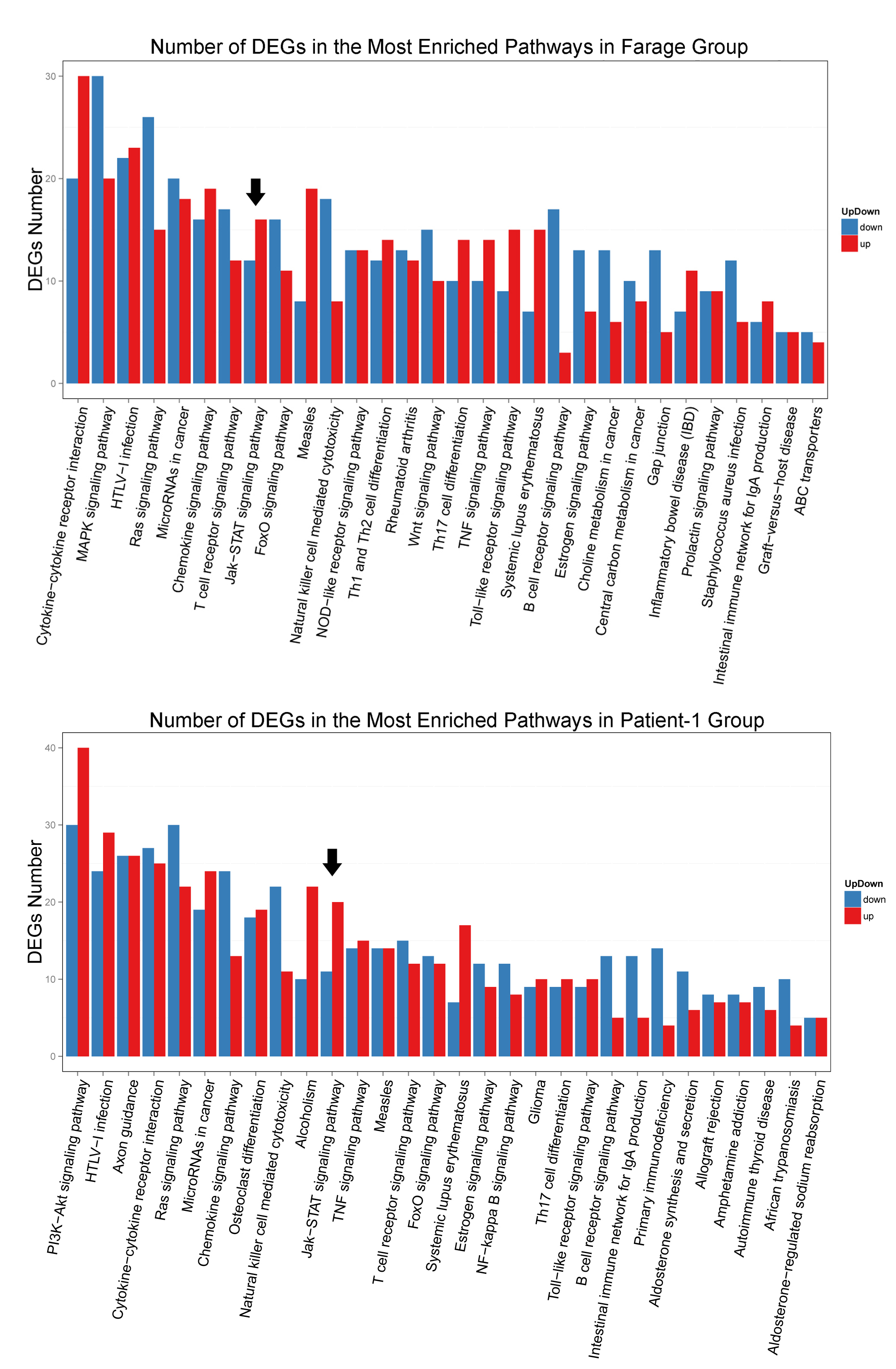
**

**Figure S2.** Pathway enrichment analysis of differentially expressed genes (DEGs) in EBV-positive DLBCL cells treated with IL-21. Top-ranked enrichment results for upregulated and downregulated genes in primary and Farage cells. The arrow indicates a common upregulation of the JAK-STAT signalling pathway among top-ranked pathways.

**
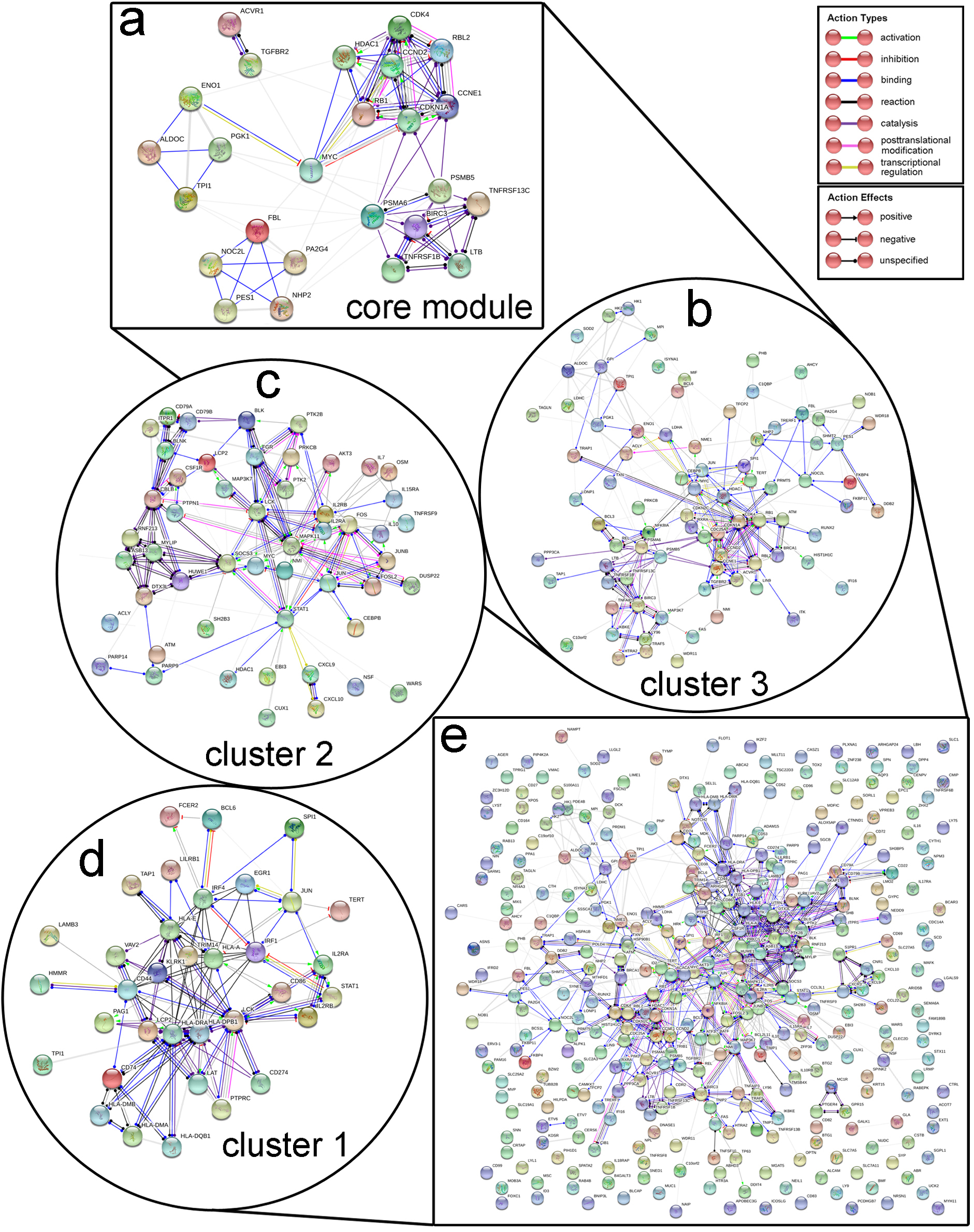
**

**Figure S3.** Molecular interaction information obtained from online database STRING. The MCODE plugin in Cytoscape was used to extract subnetworks in the entire network. Clusters 1 (**d**), 2 (**c**) and 3 (**b**) were generated from the entire picture (**e**), and the core module (**a**) was extracted from cluster 3 (**b**) by disabling the Fluff function in MCODE.

**
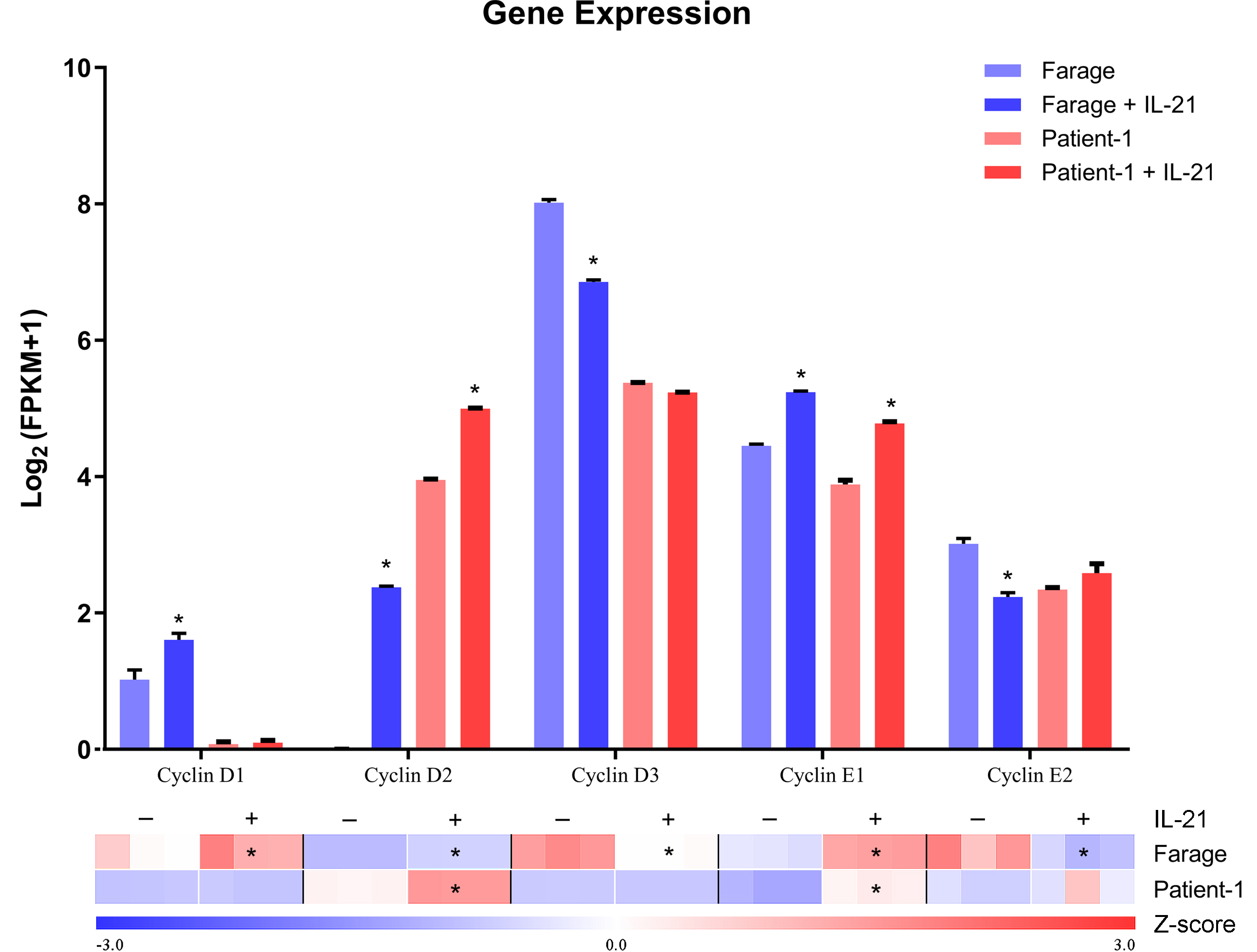
**

**Figure S4.** Subtype specific analysis of differential gene expression of cyclin D and E. Log2-(FPKM+1) data from the RNA-seq analysis were displayed as the means ± SEMs (n = 3). Z-score normalized log2-(FPKM+1) of cyclin D and E gene expression are shown in the heatmap. Statistical analysis was performed using FDR-based q values; *q < 0.01.


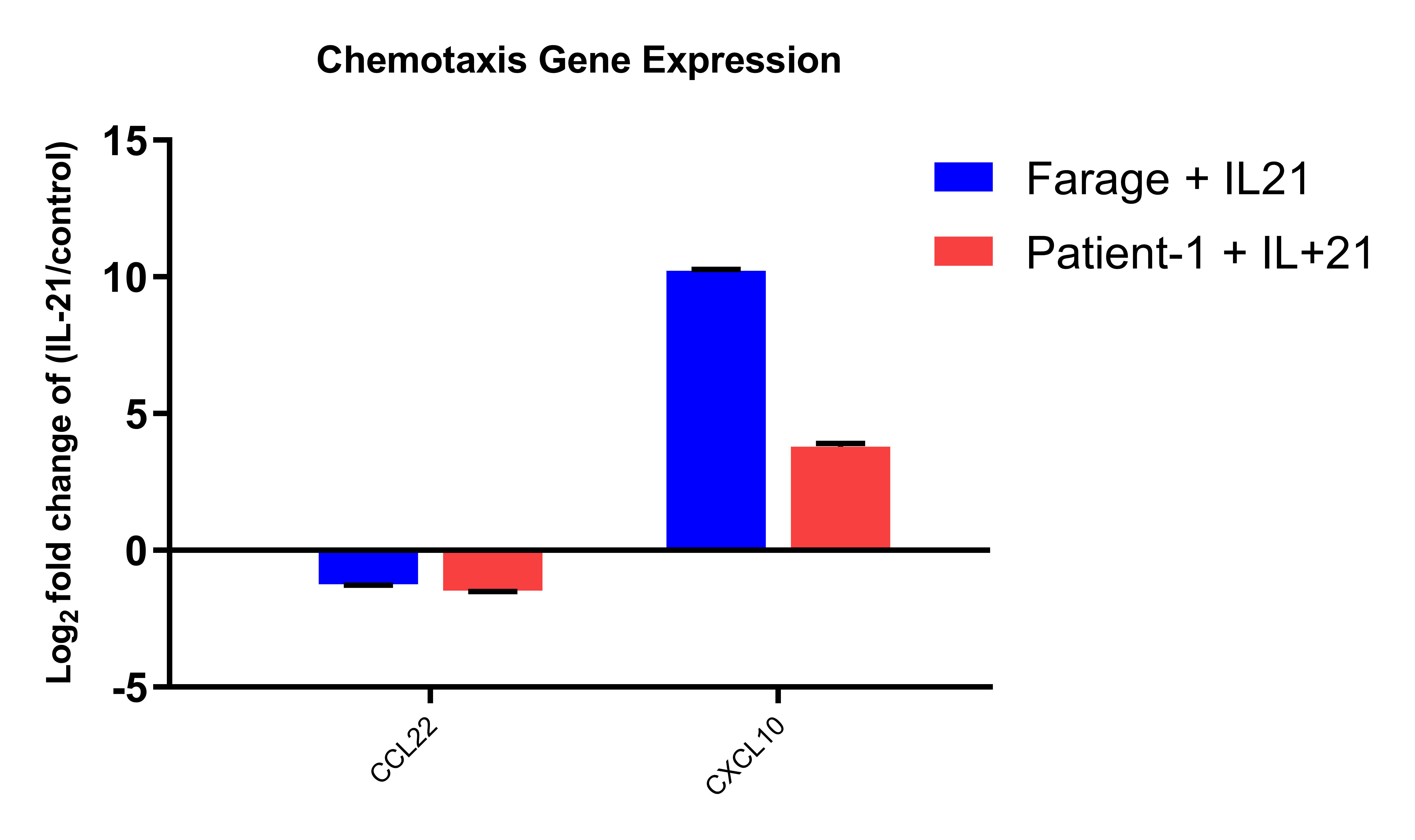


**Figure S5.** RNA-seq analysis of chemotaxis gene expression. Fold change expression of the indicated chemotaxis genes from the chemokine signalling pathway (KEGG pathway: hsa04062) in Farage cells and the primary clinical sample treated with IL-21 (100 ng/ml for 48 h) compared with the untreated samples (control) measured by RNA-seq. The log2 fold changes in gene expression values are displayed as the means ± SEMs (n = 3). Statistical analysis was performed using FDR-based q values, and all data shown were significant with a q value of < 0.01.
